# Research and Application of the Polyene Macrolide Antibiotic Nystatin

**DOI:** 10.3390/molecules31020330

**Published:** 2026-01-19

**Authors:** Xiaofeng Liu, Jiamin Zhuo, Zherui Chen, Yao Zhang, Wei Jiang, Rongfa Guan

**Affiliations:** 1 College of Food Science and Technology, Zhejiang University of Technology, Hangzhou 310014, China; 2 Zhejiang Zhenyuan Pharmaceutical Co., Ltd., Shaoxing 312088, China; 3 School of Biological and Chemical Engineering, Zhejiang University of Science and Technology, Hangzhou 310023, China

**Keywords:** nystatin, nystatin A_1_, polyene macrolide antibiotic, antifungal, biosynthesis

## Abstract

Nystatin is a polyene macrolide antibiotic with broad-spectrum antifungal activity and serves as a key therapeutic agent for superficial fungal infections. This review systematically elaborates on its multicomponent chemical nature, its mechanism of action targeting ergosterol, and highlights the potential adverse effects, such as cardiotoxicity, associated with impurities like RT6 (albonoursin). The fundamental analytical techniques for quality control are outlined. Furthermore, the clinical applications and combination therapy strategies of nystatin in treating oral diseases, vaginitis, and otitis externa are summarized in detail. Regarding biosynthesis, the assembly mechanism of nystatin A_1_ via the type I polyketide synthase pathway and its subsequent modification processes are thoroughly discussed. Emphasis is placed on the latest advances and potential of gene-editing technologies, particularly CRISPR/Cas9, in the targeted knockout of genes responsible for toxic components and in optimizing production strains to enhance nystatin yield and purity. Finally, this review prospects the future development of nystatin towards improved safety and efficacy through structural optimization, innovative delivery systems, and synthetic biology strategies, aiming to provide a reference for its further research and clinical application.

## 1. Introduction

Microbial secondary metabolites, characterized by their distinctive structures, diverse bioactivities, and wide-ranging sources, are vital resources for the advancement of therapeutic antibiotics [1]. Agents including macrolides (erythromycin), β-lactams (penicillin), aminoglycosides (streptomycin), and glycopeptides (vancomycin) have proved crucial in combating numerous bacterial illnesses [2]. Subsequent to the golden era of antibiotic discovery from 1940 to 1960, there was a notable decline in the rate of development, while the increasing menace of pathogenic drug resistance has intensified the demand for novel antibiotics [3]. During this period, semisynthesis—the chemical modification of natural products—emerged as the principal method for developing new antibiotics. Nonetheless, it proved beneficial exclusively for a restricted range of scaffold types, comprising cephalosporins, penicillins, quinolones, and macrolides. Moreover, the intricate architecture surrounding natural products sometimes hinders site-selective alteration or offers only restricted changeable regions, rendering semisynthetic methods increasingly inadequate [4,5]. Consequently, innovative discovery paradigms and methodologies are essential to acquire compounds with exceptional skeletal structures [6].

Polyene antibiotics, also known as polyene macrolide antibiotics (PEMs), are antifungal drugs distinguished by conjugated double bonds and produced by particular actinomycetes and fungi [7]. The primary representations are amphotericin B, nystatin and mandeliopepin [8]. Amphotericin B is a quintessential polyene macrolide antibiotic employed for severe systemic fungal infections [9]. Owing to its high toxicity, it must be administered intravenously, while renal function and electrolytes are monitored to mitigate side effects [10]. Nystatin is structurally similar to amphotericin B, and its biosynthesis-related gene clusters are significantly conserved [11]. Intravenous liposomal nystatin treatment maintains 60% of Candida infections resistant to amphotericin B or fluconazole within the susceptible range, demonstrating equivalent efficacy against candidemia as well as Aspergillus and Cryptococcus infections [12].

**Figure 1:**
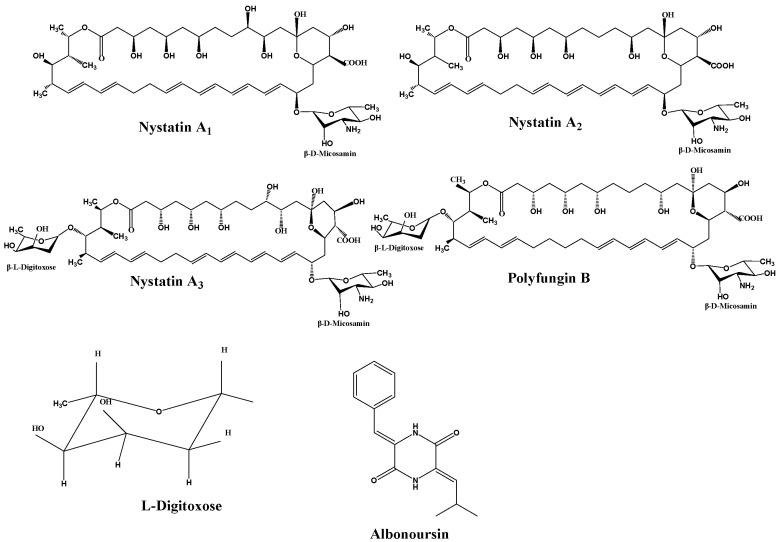
Structural formulas of components.

Nystatin is a polyene macrolide antibiotic consisting of 38 members, whose chemical formula is C_47_H_75_NO_17_. The extensive macrocyclic lactone core has three to seven conjugated double bonds, imparting distinctive UV maxima and antifungal properties [13]. The structure also includes an exocyclic carboxyl group, a mycosamine molecule, and a hemiketal moiety. In 1950, Hazen and Brown initially extracted nystatin from cultures of the soil bacterium *Streptomyces noursei*. Simultaneously, researchers in China extracted a similar compound from *Streptomyces* sp. (Norse actinomycetes) sourced from Guangdong Province, designating it as nysfungin [14]. Foreign nystatin mostly consists of nystatin A_1_, a variant that exhibits high efficacy against many fungi, including Candida albicans and Aspergillus species. It exhibits minimal cardiotoxicity in clinical applications, rendering it appropriate for the treatment of localized and gastrointestinal mycoses. Chinese nystatin consists of nystatin A_1_ (2.12–15.56%), nystatin A_3_ (27.22–53.52%), Polyfungin B (19.13–39.83%), and an undetermined component RT6 (~10%) [15] (Figure 1).

In the first stages of development, domestic researchers sought nystatin as a generic alternative to “nystatin”. Further analyses indicated significant compositional differences between the Chinese product and worldwide nystatin. Thus, the domestic formulation was named “nysfungin” to differentiate it from the reference chemical. The distinguishing characteristics are encapsulated in Table 1.

**Figure 2:**
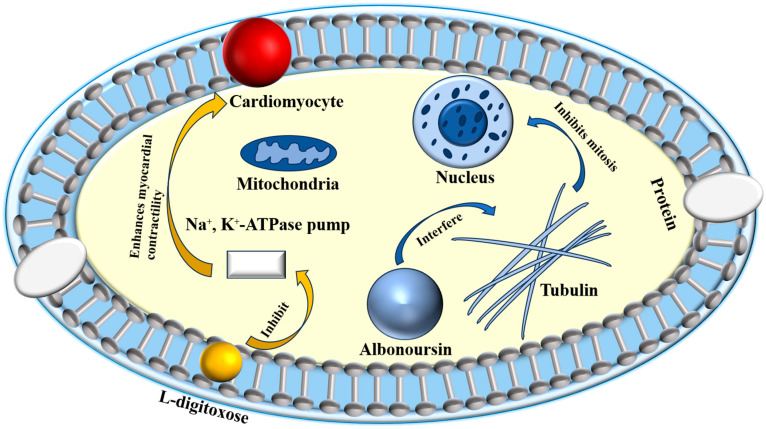
Toxic mechanisms of L-digitoxose and albonoursin.

Research has identified the previously unknown component RT6 as albonoursin, a chemical initially extracted from nystatin, which has subsequently been shown to exhibit no antifungal action [18]. Nystatin A_3_ and Polyfungin B both possess the structural motif L-digitoxose [19]. Digitoxose inhibits the Na^+^, K^+^-ATPase pump in cardiomyocytes, hence augmenting contractile force (positive inotropy). Concurrently, its negative chronotropic and dromotropic effects reduce heart rate and obstruct atrioventricular conduction [20]. These properties underlie its clinical utility in congestive heart failure. Digitalis glycosides possess a narrow therapeutic index and significant inter-individual variability, requiring stringent therapeutic medication monitoring to prevent digitalis toxicity, which can lead to a life-threatening arrhythmogenic condition [21]. It has been postulated that the L-digitoxose moiety within nystatin A_3_ and Polyfungin B may confer analogous cardiotoxic liability, precipitating dysrhythmias. Consequently, heightened vigilance is mandated during clinical use, particularly in patients at elevated risk for digitalis toxicity; proactive surveillance and readiness to manage cardiac adverse events are essential to ensure patient safety. Albonoursin is an antibacterial and antitumor dehydrocyclic dipeptide compound that belongs to the class of 2,5-diketopiperazine derivatives [22]. Research indicates that 2,5-diketopiperazines inhibit tubulin polymerization, interfere with proper mitosis, and exhibit fatal consequences [23]. This toxicity may correlate with persistent cell destruction and could potentially encourage excessive proliferation, suggesting a possible connection to malignant transformation. Structural analysis demonstrates that both amino acid residues in albonoursin experience α, β-dehydrogenation, hence augmenting its cytotoxic potency [24]. While no research has conclusively proven toxicity in humans, clinical caution necessitates that medications containing albonoursin be administered judiciously to those seeking conception or who are pregnant, to mitigate potential reproductive hazards (Figure 2).

Despite its relatively considerable toxic side effects, nystatin remains one of the most essential antifungal agents in clinical practice due to its potent, broad-spectrum activity and low resistance rate [25]. Notwithstanding its significant toxic side effects, nystatin is a crucial antifungal drug in clinical practice owing to its powerful, broad-spectrum efficacy and low resistance rate [26,27,28]. Progress in biosynthetic research and the advent of gene-editing technologies such as CRISPR/Cas9 have facilitated the execution of targeted gene knockouts to eliminate detrimental components in nystatin and its derivatives, thereby improving the yield of nystatin A_1_ [29]. These advancements create new opportunities for the manufacturing, optimization, and utilization of these antifungal drugs.

**Figure 3:**
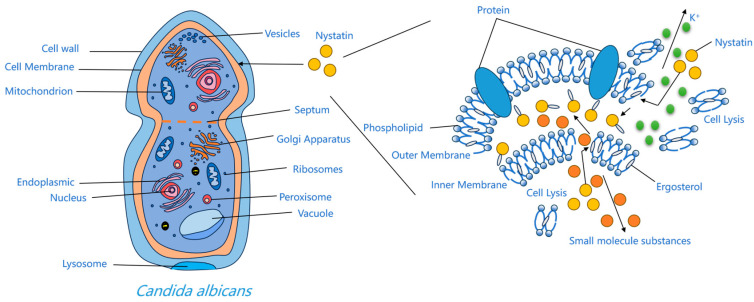
Mechanism diagram of action of nystatin A_1_.

The interaction between nystatin A_1_ and ergosterol in fungal cell membranes arises from the higher number of double bonds in its ring structure compared to cholesterol in human membranes, leading to relative selectivity [30]. This relative selectivity enhances its antifungal activity, although it also accounts for the associated hemolytic and nephrotoxic side effects [31]. Upon binding to ergosterol, the drug molecule induces size-selective transmembrane pores in the membrane, resulting in osmotic imbalance and significantly increasing permeability to small solute molecules while having little effect on larger ones [32]. These channels result from hydrophobic interactions between the polyene chain and membrane sterols, undermining the barrier and electrochemical gradients. Consequently, potassium and other essential ions are lost, glycolysis is impaired, and the cell perishes [33,34]. The thermodynamically optimal arrangement entails the hydrophobic tail directly interacting with ergosterol in the lipid membrane, and the hydrophilic chain is oriented towards the aqueous channel environment [30]. The pore-forming concept continues to be the primary explanation for the activity of polyenes. Alternative concepts—half-pore, surface adsorption, and the sterol-sponge hypothesis—have been suggested; nevertheless, the sterol-sponge model remains devoid of definitive pharmacological and toxicological validation [35]. Furthermore, oxidative stress induced by polyenes, leading to DNA damage, protein carbonylation, and lipid peroxidation, has been suggested to promote fungal cell death [36]. Regardless of the approach, binding to ergosterol is the essential prerequisite for the efficiency of polyene antifungals [30] (Figure 3).

Nystatin A_1_ demonstrates significant efficacy against *Candida* spp., notably suppressing Candida albicans, *Aspergillus* spp., Mucorales, and Blastomyces dermatitidis. Nystatin A_1_ also demonstrates therapeutic efficacy against specific protozoal or parasitic diseases [37]. It is utilized clinically for superficial candidiasis, including gastrointestinal candidiasis and vaginitis, as well as for leishmaniasis [38,39,40], demonstrating favorable outcomes in combination regimens [41,42]. Formulations containing nystatin A_1_ are available in the forms of powders, suppositories, ointments, pills, and capsules, taken orally, topically, or via irrigation. Contemporary research concentrates on nano-capsule hydrogels [43], liposomes, and alginate microparticles [44] to expand its delivery options and clinical utility.

Nystatin, a secondary metabolite derived from *Streptomyces*, is manufactured industrially at a cost that is inversely related to the titer attained by the generating strain. Classical strain enhancement depends on successive cycles of random mutagenesis combined with high-throughput screening, succeeded by statistical optimization of medium composition and fermentation conditions [45,46,47]. The comprehensive understanding of the nystA biosynthetic gene cluster and its transcriptional circuitry has rendered rational genetic engineering the primary method for yield increases. Cluster-embedded, pathway-specific regulators modulate flow by interacting with the promoters of biosynthetic genes. In nystatin, only a limited number of regulators, such as the TetR-type activator NysRI, are identified, and their overexpression often increases output by less than twofold [48]. In contrast, pleiotropic regulators (γ-butyrolactone receptors, two-component systems) and quorum-sensing signaling networks that synchronize morphological differentiation with secondary metabolism have significantly more potential for simultaneously enhancing cellular fitness and nystA expression [49]. Nystatin, at the metabolic level, is a type I polyketide whose backbone assembly is driven by malonyl-CoA and methylmalonyl-CoA. The existence of numerous biosynthetic gene clusters vying for the same precursors creates a significant bottleneck in precursor availability. Thus, intracellular overproduction of CoA esters, external supplementation of small-molecule precursors, or the deletion of essential genes in competing pathways can significantly alleviate carbon shortage. In *Streptomyces* hygroscopicus, the deletion of the tetramycin activator ttmRIV or the PKS gene ttmS1 eliminates tetramycin production. The deletions increase nystatin titers by 1.1-fold and 10-fold, respectively, offering direct evidence that eliminating a competitive pathway enhances the targeted product [50,51].

## 2. Determination of Nystatin Components

Nystatin is a multifaceted antibiotic; first assays depended on microbiological potency assessment, a process encumbered by numerous steps, extended incubation, and various causes of variability. Subsequently, ultraviolet-visible spectrophotometry (UV-Vis) was employed. However, this method is hindered by inadequate specificity and a limited quantitative range, preventing precise quantification of deleterious congeners. Currently, high-performance liquid chromatography (HPLC) is the preferred approach, providing exceptional specificity, precision, and an extensive linear dynamic range that enables dependable resolution and quantification of nystatin components. A comparative overview of these techniques is provided in Table 2. Due to strain selection, maintenance, and minor process variations potentially causing fluctuations in measured content, rigorous management controls are essential during normal manufacturing.

### 2.1. Improvement of Microbial Assay Method

The standard procedure for determining nystatin content predominantly uses the microbiological assay. This method employs the diffusion characteristics of antibiotics in agar media, assessing the widths of inhibition zones produced by standard and test samples against susceptible microbes to determine sample potency. Nystatin, a polyene antibiotic, effectively inhibits fungal growth, facilitating quantitative investigation of its antifungal effectiveness against particular fungal strains. The tube-disk approach has many significant limitations: procedural complexity necessitating multiple technical steps, extended incubation durations, and indistinct inhibition zone boundaries when employing Saccharomyces cerevisiae as the test organism. These variables combined undermine measurement accuracy and render the approach insufficient for modern hospital preparation quality control, which necessitates both precision and swift turnaround times [52].

To address these limitations, investigators have introduced a series of refinements to the conventional assay. Shen enhanced the measurement of nystatin and its tablets by altering the medium formulation, employing the strain ATCC 9763, and establishing the antibiotic concentration range at 10–50 U·mL^−1^ [53]. The phosphate buffer was adjusted to pH 6.0, and incubation occurred at 28 °C for 22 h. The revised methodology furnishes the test organism with sufficient carbon and nitrogen substrates and delivers crucial enzymatic activators for microbial metabolism. As a result, it generates distinctly defined, stable inhibitory zones devoid of secondary fronts, and zones at both elevated and diminished concentrations may be quantified instrumentally. Wang et al. developed a more efficient potency assay for polyene antibiotics by modifying the test organism, medium composition, incubation temperature, and duration [54]. This method improves reliability and operability and has been established as the standard calibration procedure for the batch replacement of the national nystatin reference standard.

The enhancements to the traditional microbiological potency assay have yielded significant results. Post-optimization, the inhibition zones display distinctly defined borders that facilitate exact instrumental measurement, resulting in correct outcomes and offering a more efficient and dependable technical method for nystatin quantification.

### 2.2. Ultraviolet-Visible Spectrophotometry

UV-Vis is extensively utilized for nystatin quantification because of its operational simplicity, speed, and satisfactory accuracy. Nystatin demonstrates peak absorption at 304 nm or 319 nm; assessing absorbance at this specific wavelength facilitates the quantification of its concentration [55]. The process involves the production of standard and test solutions with known concentrations, succeeded by spectrophotometric measurement at the maximum absorption. The absorbance ratio of the standard to the sample is utilized to determine the concentration and content of nystatin.

A multitude of researchers have corroborated and enhanced this methodology. Li et al. measured nystatin in nystatin oil at 304 nm, demonstrating exceptional linearity within the range of 7.2–16.8 μg mL^−1^ [56]. The average recovery was 100.131% ± 0.871% with a relative standard deviation of 0.870%, validating the method’s simplicity, efficiency, and high reproducibility. Ibrahem and Rashid presented two spectrophotometric techniques for pure nystatin and its formulations [57]. A method involves the condensation of vanillin with alkali to produce a yellow Schiff base. The alternative method oxidizes nystatin using an excess of bromate-bromide in an acidic medium, thereafter reacting the residual oxidant with crystal violet to produce a blue chromophore. Mohamed et al. presented an innovative green spectrophotometric method—Fourier self-deconvolution (FSD)—for the quantification of nystatin in both bulk and dose forms [58]. The method employs a straightforward mathematical transformation on the excipient-masked zero-order spectrum. A built-in Fourier wavelet performs deconvolution, and nystatin’s deconvoluted amplitude is quantified at 320 nm. A linear range of 1–25 μg·mL^−1^ was established; the method is straightforward, expeditious, and suitable for nystatin testing in pharmaceutical and laboratory formulations. Jiang formulated nystatin ointment utilizing Simple Ointment No.2 as the basis and quantified the medication UV-Vis according to protocol [59]. The stability assessment indicated a λmax of 304 nm, a mean recovery of 100.67% (RSD 0.56%), and approximately three months of stability under refrigeration. Zeng et al. noted that while analyzing nystatin vaginal pills by UV-Vis with methanol as the solvent and without filtration, the recovery rate surpassed 98% [60]. Lemus Gallego and Pérez Arroyo integrated UV-Vis with derivative and multivariate calibration techniques to concurrently quantify hydrocortisone, nystatin, and oxytetracycline in synthetic and pharmaceutical formulations [61]. The methods demonstrated excellent linearity, accuracy, and precision without necessitating a separation step, making them appropriate for the quantitative analysis of complex mixtures.

UV-Vis provides operational simplicity, speed, and minimal instrumental demands, rendering it appropriate for swift screening and initial quantification. Nonetheless, its specificity is rather low, and interference from other components is probable; hence, the approach is most effectively utilized on highly pure materials or for preliminary investigations.

### 2.3. High-Performance Liquid Chromatography

HPLC is a very effective separation method that utilizes variations in partition coefficients between the stationary and mobile phases. Upon dissolution in the mobile phase, the sample is propelled through a column filled with the stationary phase; due to the unique partition coefficients of each component, their migration velocities vary, resulting in separation. A C18 column is commonly utilized, with the mobile phase including acetonitrile and ammonium dihydrogen phosphate. Qualitative and quantitative investigations of nystatin components are conducted by evaluating retention duration and peak area [62].

HPLC has been widely utilized for the analysis of nystatin. Li et al. utilized high-performance liquid chromatography to quantify nystatin concentration and assess the stability of nystatin liniments formulated in various carriers [63]. Liniments were composed of 0.9% sodium chloride, 2.5% sodium bicarbonate, glycerol suppository solution, and pure glycerol, with each group further split for observation. The results indicated that the 2.5% sodium bicarbonate formulation was unstable, whereas the other three vehicles exhibited negligible content variation over four weeks and were interchangeable. Consequently, a 2.5% sodium bicarbonate solution is inappropriate as a substrate for nystatin liniment. Abdelrahman et al. isolated nystatin by HPLC on a C8 column utilizing isocratic methanol–0.05% SDS (40:60, *v*/*v*) at a flow rate of 0.8 mL·min^−1^, 25 °C, and detection at 220 nm [64]. Linearity was confirmed within the ranges of 5–50, 4–50, and 4–40 µg·mL^−1^, with nystatin eluting at 3.52 min.

Elsharkawy et al. improved the HPLC method for the concurrent quantification of nystatin in industrial effluent samples through an experimental design approach [65]. A full-factorial screening design was utilized for chromatographic variables, succeeded by optimization through a central composite design, to determine the ideal circumstances for achieving maximum resolution between adjacent peaks within a runtime of under 5 min. The experimental findings revealed a linear range of 1.00–25.00 μg·mL^−1^ for nystatin, and the proposed methodology was effectively utilized for the measurement of the target pharmaceutical chemicals in rinse wastewater samples. High-Performance Liquid Chromatography–Mass Spectrometry (HPLC-MS) integrates the separation proficiency of HPLC with the heightened sensitivity and selectivity of mass spectrometry, facilitating accurate identification and quantification of constituents in nystatin. Chang et al. utilized HPLC-MS to determine the structural components of nystatin and to estimate their possible toxicity [17]. The experimental results demonstrated that the primary components and their concentrations varied between the two. Nystatin, nystatin A3, and Polyfungin B possess the structural motifs L-digitoxose, perhaps linked to cardiotoxicity, and 2,5-diketopiperazine, which may influence reproduction.

HPLC facilitates the effective separation of several components of nystatin, such as nystatin A_1_, A_3_, and Polyfungin B, enabling the investigation of intricate matrices. However, in comparison to UV spectrophotometry, it incurs greater expenses due to the necessity for specialized equipment and skilled workers. Despite this limitation, HPLC provides significant benefits in compositional analysis, quality control, and stability studies of nystatin, offering crucial technical assistance for the development and implementation of nystatin-related products.

## 3. Clinical Applications of Nystatin

### 3.1. Nystatin for the Treatment of Oral Diseases

Nystatin demonstrates antifungal properties that successfully inhibit fungal proliferation and is frequently utilized in the treatment of oral conditions. Rinsing with nystatin solution preserves oral hygiene and diminishes fungal colonization [66]. Chlorhexidine mouthwash exhibits significant antifungal effectiveness. Liu indicated that the combination of nystatin solution and compound chlorhexidine mouthwash significantly reduced candidal counts and inflammatory indicators in post-orthodontic patients with Candida [67]. This combination enhanced therapeutic efficacy compared to chlorhexidine alone.

Deng et al. assessed the efficacy of nystatin combined with honey in the prevention and treatment of chemotherapy-induced oral mucositis in patients with leukemia [68]. Honey, abundant in fructose, vitamins, and amino acids, serves as a natural sweetener, facilitates wound healing, and reduces the risk of infection. The research indicated that the amalgamation of nystatin and honey reduces both the frequency and intensity of OM, mitigates patients’ adverse feelings, and consequently possesses significant clinical promoting potential.

Yan et al. assessed the concurrent administration of nystatin tablets and Kangfuxin solution in individuals following affected third-molar extraction [69]. The findings indicated that this regimen significantly decreases the occurrence of oral infections, preserves oral microbial homeostasis, and adjusts the Th17/Treg immunological balance, hence improving patients’ antibacterial and anti-infective abilities.

Candida albicans is the primary causative agent of thrush [70], and antifungal medications like nystatin and miconazole are commonly employed as topical treatments. Studies indicate that the combination of topical fluconazole and nystatin ointment significantly surpasses the efficacy of fluconazole administered alone. The combination enhances efficacy, reduces adverse effects, shortens treatment duration, and improves outcomes in affected children [71].

Furthermore, Paiva et al. indicated that the essential oil of lemongrass (Cymbopogon citratus) demonstrates both fungistatic and fungicidal properties against oral yeasts [72]. The amalgamation of this botanical drug with nystatin exhibits potential antifungal effectiveness against isolates from oncologic patients, indicating that simultaneous administration of this phytotherapeutic preparation may augment antimicrobial results in individuals undergoing nystatin treatment.

Nystatin exhibits significant antifungal effectiveness in the management of oral illnesses. The simultaneous application of sodium bicarbonate, honey, or Kangfuxin solution enhances efficacy while reducing costs and adverse effects. Co-administering nystatin with natural remedies such as essential plant oils presents a novel treatment approach.

### 3.2. Nystatin for the Treatment of Vaginitis

Vulvovaginal candidiasis is an endogenous infection induced by Candida species, predominantly Candida albicans [73]. The prevailing clinical protocol often utilizes sequential therapy, commencing with nifuratel-nystatin vaginal soft capsules, succeeded by Lactobacillus vaginal capsules [74]. This technique demonstrates some effectiveness; nonetheless, recurrence is common, requiring additional investigation into more potent therapy strategies.

The simultaneous intravaginal application of fluconazole tablets and nystatin vaginal suppositories for vulvovaginal candidiasis (VVC) has been assessed [75]. The research indicated that the combination swiftly mitigated local symptoms and markedly improved overall therapeutic effectiveness. Further investigations indicated that a triple therapy regimen consisting of nystatin, fluconazole tablets, and Lactobacillus vaginal capsules had a 96.4% cure rate in patients with vulvovaginal candidiasis, with a mere 3.6% recurrence rate [76]. These data validate that nystatin successfully eliminates Candida species and reinstates vaginal microbial equilibrium, highlighting its essential function in the therapy of VVC.

Wang assessed the therapeutic efficacy of nifuratel-nystatin vaginal soft capsules versus miconazole nitrate suppositories in the treatment of vaginitis by analyzing oxidative stress markers, inflammatory indicators, and unpleasant effects [77]. The findings demonstrated that nifuratel-nystatin vaginal soft capsules outperform miconazole nitrate suppositories in mitigating inflammatory responses, decreasing oxidative stress, and relieving clinical symptoms, hence, justifying wider clinical usage.

Xie and Li established that the combination of nifuratel-nystatin vaginal capsules and ornidazole suppositories for trichomonal vaginitis more effectively maintains immune homeostasis, reduces inflammation, enhances microcirculation, and significantly decreases recurrence rates compared to ornidazole alone [78]. While ornidazole suppositories effectively suppress Trichomonas and anaerobic bacteria, they also quickly promote the development of resistance. Nifuratel-nystatin vaginal capsules safeguard Lactobacillus, impede microbial glycolysis, reestablish normal vaginal pH, and improve self-cleansing mechanisms. Nifuratel, a broad-spectrum antibiotic, eliminates infections and inhibits their growth; its combined use markedly enhances treatment efficacy.

Researchers have assessed the sequential protocol of Honghe Fujie vaginal wash in conjunction with nifuratel-nystatin vaginal soft capsules, succeeded by Lactobacillus vaginal capsules for the treatment of mycotic vaginitis. The results indicated that this combination swiftly alleviates clinical symptoms, regulates vaginal micro-ecology, decreases CRP and PCT levels, minimizes recurrence rates, and demonstrates a high safety profile, thus providing a novel strategy for the management of mycotic vaginitis [79].

### 3.3. Nystatin for the Treatment of Otitis

Fungal external otitis is an infectious condition resulting from fungus infiltrating the external auditory canal, with heightened susceptibility occurring when human immunity diminishes or the canal’s epidermis is compromised [80]. Clinical signs include pruritus, discomfort, aural fullness, little watery discharge, and, in severe instances, auditory impairment [81]. Pathological studies demonstrate that the condition is primarily attributable to Aspergillus, Candida, or mixed fungal-bacterial infections. Topical antifungal treatments constitute the primary treatment; frequently utilized medications include nystatin, ketoconazole, and clotrimazole. Woods and Saliba established that nystatin does not induce hearing loss or cochlear hair-cell damage in guinea pigs, hence affirming its safety [82]. Furthermore, it has been documented that oral nystatin suspension, when provided biweekly, can effectively treat fungal external otitis, with observable improvement within three weeks [83].

Clotrimazole ointment, a commonly utilized antifungal, is effective against Candida, Cryptococcus, and Aspergillus species in fungal external otitis. The constricted, innervated canal makes cotton-bud application unpleasant, reduces compliance, and leaves hidden foci unaddressed, promoting recurrence. Nystatin powder, administered via insufflation, prevents direct touch, enhances coverage, eradicates untreated niches, and increases both compliance and efficacy. Yang established that endoscopic insufflation of nystatin powder results in significant mycological clearance, minimal recurrence, substantial reduction in inflammation, and rapid symptom alleviation, all while maintaining safety and simplicity [84]. Wang et al. noted favorable results when nystatin cream was uniformly administered to the canal, succeeded by 2% salicylic-acid alcoholic drops once symptoms diminished [85].

**Figure 4:**
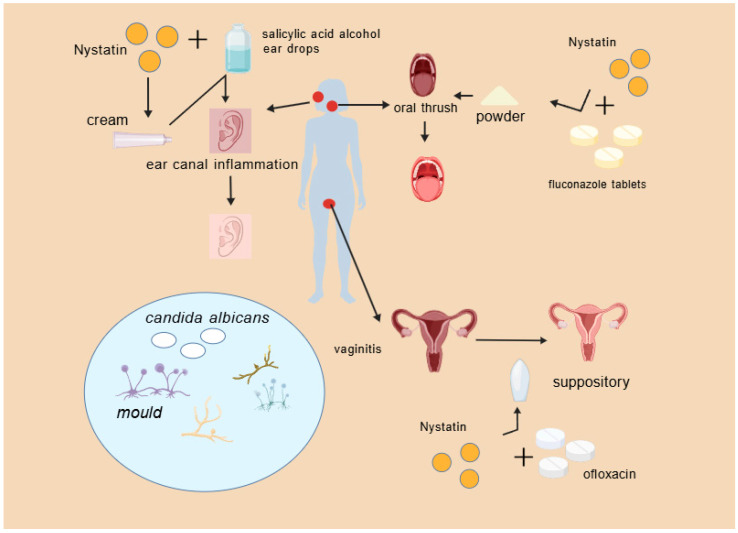
Nystatin combination drug scenario.

Nystatin powder provides specific benefits in treating fungal external otitis, especially when administered with otoscopic supervision, thus improving therapeutic effectiveness and patient adherence (Figure 4).

## 4. Biosynthesis of Nystatin

### 4.1. The Biosynthetic Process of Nystatin

**Figure 5:**
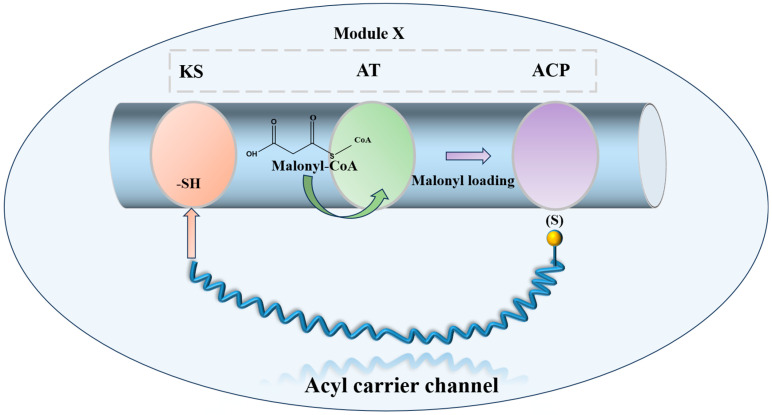
Schematic diagram of the minimal functional unit of Type I PKS extension module.

Combinatorial biosynthesis is a research approach that generates structural variety in natural products by substituting, augmenting, or altering biosynthetic genes. Progress in polyketide synthase (PKS) mechanisms has stimulated enzyme reengineering approaches. This involves inserting or deleting PKS modules to modify chain length, exchanging modules or domains to alter building blocks, and customizing domains, such as β-keto reductases, to adjust skeleton decorating. All these methodologies seek to generate additional molecules with innovative scaffolds, thus augmenting the repository for the identification of novel antibiotic agents. The polyene macrolide antibiotic nystatin A_1_ is synthesized by a type I PKS with a modular structure that includes the standard β-ketoacyl-acyl carrier protein synthase (KS), acyltransferase (AT), and acyl carrier protein (ACP) domains [86,87] (Figure 5).

**Figure 6:**
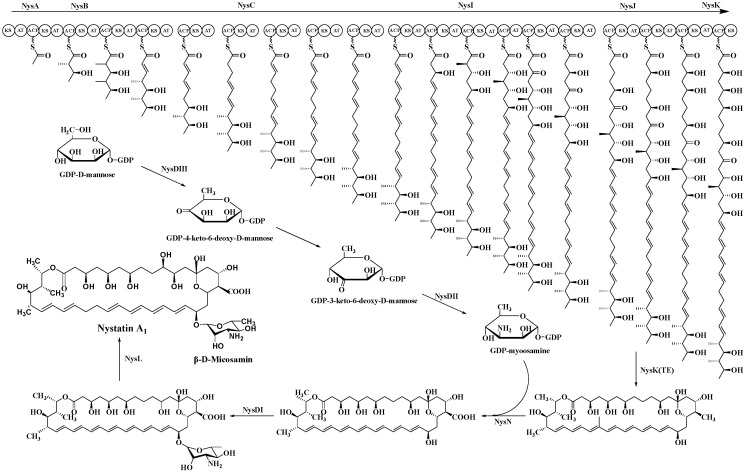
Biosynthesis pathway of nystatin.

In A_1_ biosynthesis, the AT domain of the loading module NysA selectively loads malonate onto the neighboring ACP, followed by decarboxylation catalyzed by KS, which produces the initial acetyl unit. The elongation modules then perform Claisen-type condensations that integrate fifteen malonyl-CoA and three methylmalonyl-CoA extender units. The thioesterase (TE) domain facilitates the release of the macrolactone core, subsequently allowing tailoring processes to transform the linear polyketide into nystatin A_1_ [88]. Post-PKS alterations include mycosamine glycosylation, hydroxylation at C-10, and the oxidation of the C-16 exocyclic methyl to a carboxyl group [89,90,91] (Figure 6).

The trehalose amine glycosylation of nystatin A1 is regulated by NysDIII, NysDII, and NysDI, occurring at the C19 position. The first stage of glycosylation involves the enzyme GDP-mannose-4,6-dehydratase, encoded by NysDIII, which catalyzes the transformation of GDP-mannose into GDP-4-keto-6-deoxymannose. Thereafter, through the action of an isomerase, the molecule autonomously converts to 3-keto-6-deoxy-GDP-mannose. Subsequently, facilitated by NysDII, an amidation reaction transpires, resulting in the synthesis of GDP-trehalose amine. The trehalose amine is ultimately linked to the macrolide backbone via a deoxy sugar component, facilitated by the glycosyltransferase encoded by NysDI. Cytochrome P450 monooxygenases catalyze the carboxylation, epoxidation, and hydroxylation of the polyene macrolide polyketide backbone. The P450 monooxygenases encoded by NysN and NysM facilitate the transfer of electrons from NADH, converting the oxidation group at the C16 position to a carboxyl group, whereas NysL catalyzes the hydroxylation at the C10 position.

### 4.2. Combinatorial Biosynthesis of Nystatin

Researchers employed combinatorial biosynthesis to focus on three specific areas of nystatin: the C-28/C-29 polyene segment, the C-16 exocyclic carboxyl group inside the macrolactone, and the C-5/C-7/C-9/C-10 polyol domain, resulting in a variety of optimal derivatives.

Brautaset et al. investigated the biosynthesis mechanism of nystatin through polyketide synthase and designed a sequence of biosynthetic domains within this pathway [92]. Researchers initially deleted the C-terminal region of the KR domain in module 4 of NysC, the ACP domain, and the entirety of module 5, resulting in the mutant strain ERD48, which synthesized the hexaene nystatin derivative S48HX. This approach markedly diminished both yield and biological activity. Through the examination of ER5 domain inactivation inside the NysC module, the ERD44 mutant strain was generated, resulting in the heptaene nystatin derivative S44HP. This chemical exhibits superior solubility, and experimental findings indicate that its biological activity significantly exceeds that of nystatin [93]. Researchers demonstrated that two amino acid changes in the ER5 domain of the NPP PKS NppC transform the heptaene core, resulting in the new antibiotic NPPB1 with significantly enhanced pharmacokinetics [94]. In the polyol sector of S44HP, the C-5 or C-7 carboxyl groups were substituted with keto functions [95]. Targeted alterations were incorporated into KR17 and KR16 of NysJ, resulting in BSG013 and BSG017, respectively. In comparison to S44HP, BSG013 showed a two-fold reduction in antifungal activity and a one-fold drop in hemolytic activity, whereas BSG017 displayed a four-fold decrease in antifungal activity and a one-fold reduction in hemolytic activity. Researchers later altered the NysN domain by substituting the carboxyl group with a methyl group at position C16, resulting in BSG020 and BSG031. These compounds exhibited improved antifungal efficacy and less hemolytic toxicity; however, their exceedingly low yields significantly constrained more testing. Brautaset et al. substituted the histidine in the DH15 module of PKS NysJ, resulting in the mutant NJDH15 [95]. The C-5/C-7 keto-analogues BSG013/BSG017 exhibited diminished activity and reduced hemolytic potential, while the C-9/C-10 hydroxyl-modified BSG002 demonstrated significant declines in both activity and titer. In the C-16 carboxyl region, the mutant 16-DecNys exhibited reduced hemolysis but insufficient production. The C16 methyl position in the structure of S44HP was replaced with a carboxyl group, resulting in the formation of 16-decarboxy-16-methyl-28,29-dihydroxynystatin (BSG005). In comparison to S44HP, BSG005 exhibits a notable enhancement in yield, attaining 40% of that of S44HP, accompanied by a 1.5-fold decrease in hemolytic activity. In comparison to the analogous product 16-DecNys, the yield of BSG005 has increased twentyfold. BSG005 demonstrates antifungal activity that is twice that of S44HP and twenty times that of nystatin, suggesting that the medication retains significant antifungal efficacy while minimizing hemolytic effects. The augmented yield additionally facilitates further investigation and application.

**Figure 7:**
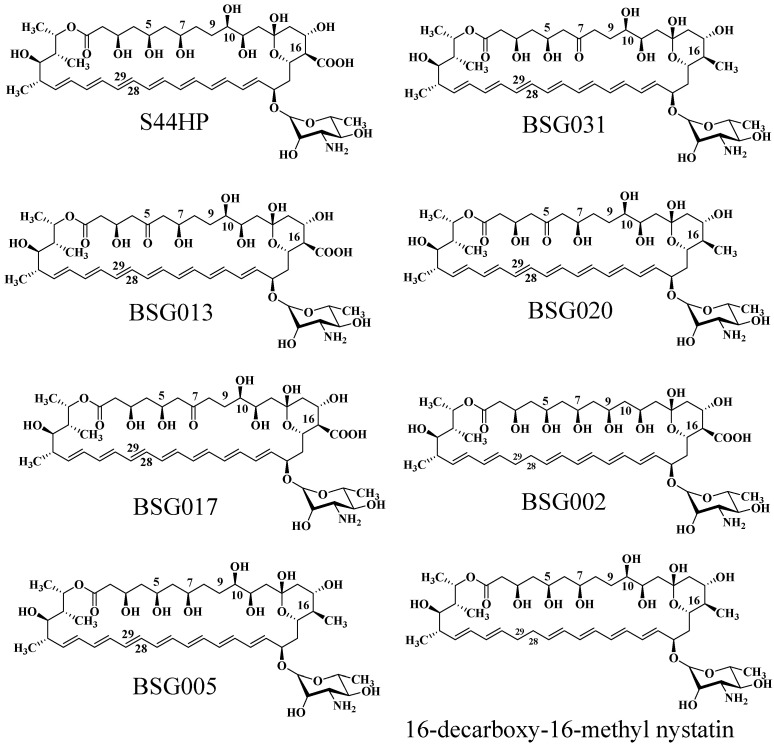
The molecular structure of natamycin analogs produced through biosynthetic engineering.

Researchers inactivated the nysL cytochrome P450 gene inside the nystatin biosynthetic gene cluster, successfully producing 10-deoxynystatin [89]. The MIC50 of this compound against Candida albicans was assessed, revealing that its inhibitory efficacy is equivalent to that of nystatin, suggesting that the hydroxyl group at the C10 position does not substantially influence nystatin’s activity against Candida albicans. Consequently, Brautaset et al. altered the nysL gene and incorporated it into the S44HP fermentation strain, resulting in the synthesis of a novel compound, BSG022 [95]. In comparison to S44HP, BSG022 had comparable biological activity; however, its HC50 value was reduced, signifying an enhancement in hemolytic toxicity. This result indicates that the hydroxyl group at the C10 position has a negligible influence on the antibacterial efficacy of the antibiotic; however, it may exert some effect on hemolysis (Figure 7).

## 5. Application of CRISPR/Cas9 Gene Editing in Streptomyces

Gene-editing technology, a pivotal domain in contemporary analytical biology, is essential for investigating gene function. In the last 15 years, gene-editing methods utilizing designed nucleases have experienced three major advancements: zinc-finger nucleases (ZFNs) [96], transcription activator-like effector nucleases (TALENs) [97], and the CRISPR/Cas system. The fundamental mechanism of these three systems is based on a DNA recognition domain and a DNA cleavage domain [98]. In contrast to the CRISPR/Cas system, ZFN and TALEN technologies are constrained in their applicability because of their intricate procedures, protracted assembly, and elevated prices.

In 2013, the CRISPR/Cas system—A groundbreaking genome-editing technology—was utilized in mammalian cells, garnering global interest [99]. CRISPR/Cas9 gene editing is a mechanism originating from the bacterial adaptive immune system that facilitates accurate genetic alteration via RNA-directed DNA cleavage. A solitary plasmid containing the sgRNA specific to the target gene, the Cas9 gene, and the homologous arms next to the target locus is inserted into *Streptomyces*. The produced sgRNA directs the Cas9 protein to cleave the target gene, facilitating gene knockout through homologous recombination with the neighboring arms [100,101]. The genome of *Streptomyces* is the largest among all known prokaryotic organisms and possesses a high GC content (typically exceeding 70%), rendering genetic manipulation of the *Streptomyces* genome more complex than that of other model organisms, such as *Escherichia coli* and *Saccharomyces* cerevisiae. The existing genetic manipulation techniques for *Streptomyces* are constrained, mostly depending on conventional site-specific recombination systems such as Cre/loxP, Dre/rox, Flp/FRT, and lnt-Xis. Nonetheless, these methodologies typically exhibit numerous issues, including intricate procedures, prolonged testing durations, and diminished efficiency. The CRISPR/Cas9 technology facilitates highly effective, locus-specific genome editing, encompassing insertion, repair, and replacement procedures [102,103].

**Figure 8:**
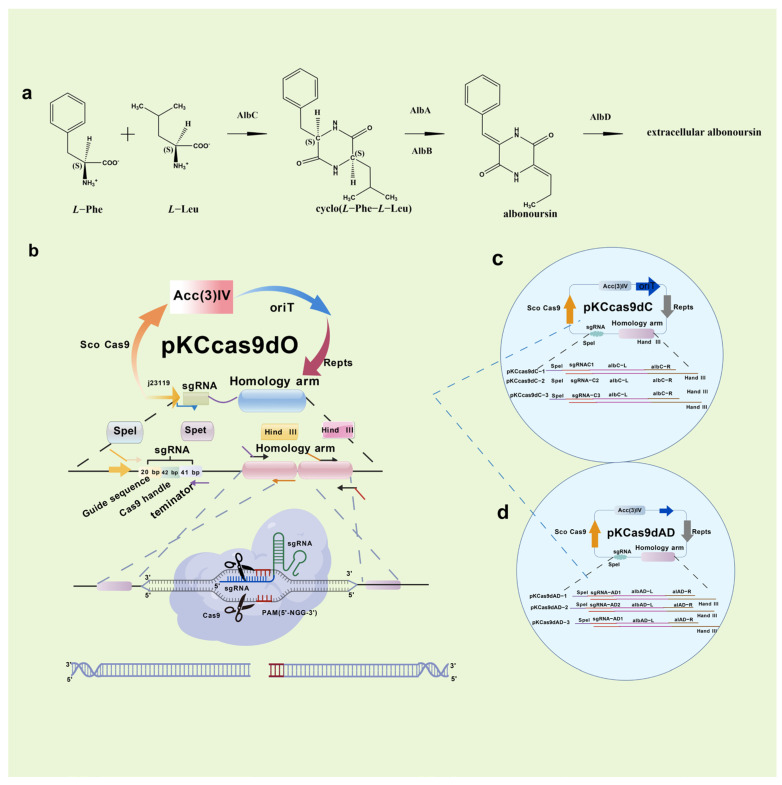
Synthesis pathway and knockout of RT6 components. *Streptomyces* sp. ZY01 in vivo RT6 biosynthesis pathway (**a**); How the CRISPR/Cas9 system knockout plasmid PKCCas9dO works (**b**); Schematic diagram of knockout plasmid PKCCas9dC (**c**); and Schematic diagram of knockout plasmid PKCCas9dAD (**d**).

In 2014, Cobb initially employed CRISPR/Cas9 in Streptomyces lividans, excising the 31 kb undecyl prodigiosin biosynthetic gene cluster [104]. He et al. subsequently created the pKCcas9dO method, attaining 60–100% efficiency for single or multi-gene knockouts [105]. The selection of sgRNA and the composition of the medium significantly affected efficiency. In 2019, Mo et al. developed pMWCas9, effectively deleting and substituting erythromycin PKS and other biosynthetic genes in Saccharopolyspora erythraea and *Streptomyces* spp. [106]. Xie et al. established a type I CRISPR/Cas methodology facilitating targeted deletions (8 bp–100 kb) with over 92% efficiency across phylogenetically distinct Streptomyces strains [107]. This technique also allows for the insertion of DNA cassettes to facilitate heterologous expression and activate dormant gene clusters, significantly enhancing *Streptomyces* research. Kim et al. altered Cas9-BD by including poly-aspartate tags, which diminished off-target binding and cytotoxicity [108]. This modified system has been utilized across many *Streptomyces* species, offering a versatile and high-efficiency editing tool for high-GC actinomycetes. The secondary metabolites of *Streptomyces* include the cytotoxic compound RT6, which is difficult to eliminate. In collaboration with Zhejiang Zhenyuan Pharmaceutical, Ai disrupted essential genes responsible for RT6 biosynthesis in the high-yield nystatin producer *Streptomyces* sp. ZY01, significantly enhancing nystatin purity [15] (Figure 8).

These examples demonstrate that CRISPR/Cas9 facilitates the convenient and efficient modification of antibiotic biosynthesis routes.

Researchers have noted that identical plasmids exhibit variable performance among *Streptomyces* species, presenting practical difficulties. Alternative Cas proteins have been assessed to overcome this constraint. Alongside the standard SpCas9, orthologues such as Sth1Cas9 from Streptococcus thermophilus, SaCas9 from Staphylococcus aureus, and FnCpf1 from Francisella tularensis have been evaluated and demonstrated to facilitate precise genome alteration with significant efficacy in streptomycetes. In 2024, Tan et al. revealed that Cas12j has superior transformation efficiency compared to SpCas9 and Cas12a, significantly enhancing editing efficacy for activating BGCs in *Streptomyces* sp. A34053, while SpCas9 and Cas12a face entry obstacles [109]. The findings suggest that Cas12j can mitigate the access constraints affecting SpCas9, thereby enhancing the applicability of CRISPR/Cas systems for genome editing in *Streptomyces*.

## 6. Prospective Directions

Invasive fungal diseases have emerged as a significant global public health concern owing to the rise of novel pathogens, heightened medication resistance, and an expanding immunocompromised demographic. Natural antifungal agents encompass polyenes, nucleosides, echinocandins, and others. Nystatin is restricted to second-line application because of its significant nephrotoxicity and narrow therapeutic index. Lipid-based formulations have been created to mitigate toxicity; nevertheless, their use is limited by elevated costs and residual toxicity [110,111]. Structural modifications have yielded analogues like BSG005 that preserve antifungal efficacy while mitigating nephrotoxicity [97], providing a more secure clinical alternative and enhancing the comprehension of the structure-activity connections of polyene antibiotics [112].

Simultaneously, advancements have been achieved in the studies into the mechanism of polyene pharmaceuticals. Historically, researchers believed that polyene pharmaceuticals eradicated fungi by creating ion channels. Recent research indicates that amphotericin B can remove ergosterol to create extramembranous sponge-like complexes, resulting in pore development and ion leakage, while also extracting and binding to cholesterol in human cell membranes, which leads to nephrotoxicity. This work offers guidance for the development of novel compounds that specifically bind to ergosterol while circumventing cholesterol binding [113,114,115]. A recent study has, for the first time, elucidated the full structure of the heptameric ion channel created by amphotericin B, providing a definitive structural template for the rational design of highly selective polyene medicines, including nystatin derivatives [116].

Advancements in gene editing and biosynthetic technologies have created novel prospects for the synthesis and enhancement of nystatin. Modern genetic engineering techniques primarily focus on inactivating the reductase domain of polyketide synthase (PKS) or modifying post-PKS genes. In the future, acquiring a profound understanding of the substrate selectivity of PKS domains may enable the modification of PKS to produce derivatives with innovative polyketide backbone architectures. Employing CRISPR/Cas9 for accurate genetic alterations of production strains—such as disrupting pathways for hazardous byproducts or augmenting precursor availability—offers potential for the high-yield, high-purity, and low-toxicity synthesis of nystatin and its derivatives. These developments will considerably augment the efficacy of this traditional medication in combating severe fungal infections.

## 7. Conclusions

Nystatin, a polyene macrolide antibiotic, displays its antifungal activity by binding to ergosterol in fungal cell membranes. It is extensively used for the treatment of localised fungal infections, including those in the mouth cavity, external ear canal, and vagina. Nonetheless, the hazardous contaminant (RT6) present in its multi-component mixture restricts its clinical safety. At present, high-performance liquid chromatography has emerged as a pivotal technique for quality control. Utilising combinatorial biosynthesis and CRISPR/Cas9 gene editing technologies, genes associated with toxic components can be eliminated to improve product purity, while alterations to polyketide synthase domains can produce derivatives that maintain antifungal efficacy with less toxicity. Subsequent research ought to persist in concentrating on the synthetic biological engineering of production strains, the creation of innovative low-toxicity derivatives, and the enhancement of distribution strategies, like liposomes and nanoparticles. This would enhance nystatin’s safety and efficacy, so it more effectively tackles the issues presented by invasive fungal infections.

## Figures and Tables

**Table 1:** Comparison of structure and physicochemical properties of domestic and international nystatin.

Property	Domestic Nystatin	International Reference Nystatin	Ref.
Structure	nystatin A_1_, A_3_, Polyfungin B, RT6	nystatin A_1_ (≥93%), nystatin A_2_, nystatin A_3_	[14,15,16,17]
pH	6.5~8.0	6.5~8.0	[16]
Toxicological properties	Cardiotoxicity, cytotoxicity, reproductive toxicity reported	No significant adverse effects recorded	[15,16,17]
Melting point	Decomposes on heating; no sharp melting point (onset 160 °C)	Decomposes > 160 °C; no clear melt	[15,16,17]
Characters	Pale-yellow, hygroscopic, odorous powder; unstable to light, air, acid or alkali	Yellow crystalline powder; similarly unstable to light, air, acid or alkali	[17]
Solubility	Slightly soluble in MeOH, EtOH, n-PrOH, n-BuOH, DMF, pyridine, glacial AcOH; insoluble in H_2_O, ether, esters, CHCl_3_, benzene	Sparingly soluble in MeOH, EtOH, acetone; insoluble in H_2_O, ether, esters, CHCl_3_, benzene	[17]

**Table 2:** Comparison of determination methods for penicillin content.

Methods	Merits	Limitations	Applications	Ref.
Microbial assay	Ecologically relevant; low running cost	Labor-intensive; susceptible to multiple confounding factors; semi-quantitative	Early-stage antibiotic potency testing	[52,53]
UV–Vis spectrophotometry	Simple protocol; inexpensive instrumentation; rapid turnaround	Poor selectivity; narrow linear range	Routine screening where high throughput outweighs accuracy	[54,55,56,57,58,59,60]
High-performance liquid chromatography	High specificity and precision; wide linear dynamic range	High instrument cost; complex method development; long analysis time	Pharmacopoeial assays, stability studies and formulation R&D	[61,62,63,64]

## Data Availability

No new data were created or analyzed in this study. Data sharing is not applicable to this article.

## Abbreviations

The following abbreviations are used in this manuscript:

HPLC	High-performance liquid chromatography
UV-Vis	Ultraviolet-visible spectrophotometry
FSD	Fourier self-deconvolution
VVC	Vulvovaginal candidiasis
PKS	Polyketide synthase
KS	β-ketoacyl-acyl carrier protein synthase
AT	Acyltransferase
ACP	Acyl carrier protein
TE	Thioesterase
ZFNs	Zinc-finger nucleases
TALENs	Transcription activator-like effector nucleases
